# SERAAK2 as a Serotonin Receptor Ligand: Structural and Pharmacological In Vitro and In Vivo Evaluation

**DOI:** 10.3390/molecules30234633

**Published:** 2025-12-02

**Authors:** Agnieszka A. Kaczor, Agata Zięba, Tadeusz Karcz, Michał K. Jastrzębski, Katarzyna Szczepańska, Tuomo Laitinen, Marián Castro, Ewa Kędzierska

**Affiliations:** 1 Department of Synthesis and Chemical Technology of Pharmaceutical Substances with Computer Modeling Laboratory, Faculty of Pharmacy, Medical University of Lublin, 4A Chodźki St., PL-20093 Lublin, Poland; zieba.agata@gmail.com (A.Z.); michal.jastrz1998@gmail.com (M.K.J.); 2 Department of Technology and Biotechnology of Drugs, Faculty of Pharmacy, Jagiellonian University Medical College, Medyczna 9, PL-30688 Cracow, Poland; t.karcz@uj.edu.pl; 3 Department of Medicinal Chemistry, Maj Institute of Pharmacology, Polish Academy of Sciences, 12 Smetna Str., 31-343 Krakow, Poland; k.szczep@if-pan.krakow.pl; 4 School of Pharmacy, University of Eastern Finland, Yliopistonranta 1, P.O. Box 1627, FI-70211 Kuopio, Finland; tuomo.laitinen@uef.fi; 5 Department of Pharmacology, Center for Research in Molecular Medicine and Chronic Diseases (CIMUS), Universidade de Santiago de Compostela, Avda de Barcelona, E-15782 Santiago de Compostela, Spain; marian.castro@usc.es; 6 Instituto de Investigación Sanitaria de Santiago de Compostela (IDIS), Travesía da Choupana s/n, E-15706 Santiago de Compostela, Spain; 7 Department of Pharmacology and Pharmacodynamics, Faculty of Pharmacy, Medical University of Lublin, 4A Chodźki St., PL-20093 Lublin, Poland

**Keywords:** ADMET studies, behavioral studies, GPCRs, molecular dynamics, molecular docking, SERAAK2, serotonin receptors

## Abstract

Serotonin receptors, in particular 5-HT_1A_ and 5-HT_2A_ receptors, are important molecular targets for the central nervous system (CNS) disorders, such as schizophrenia, depression, anxiety disorders, memory deficits, and many others. Here, we present structural and pharmacological evaluation of a serotonin receptor ligand, SERAAK2, identified in a structure-based virtual screening campaign. Molecular docking studies revealed that SERAAK2 binds with its molecular targets via Asp3.32 as the main anchoring point, which is typical for orthosteric ligands of aminergic GPCRs. Molecular dynamics simulations confirmed the stability of the ligand binding poses in the studied receptors. MMGBSA calculations were in accordance with the receptor in vitro binding affinity studies, which indicated that SERAAK2 is a potent ligand of 5-HT_1A_ and 5-HT_2A_ receptors. It was also found that SERAAK2 displays favorable ADMET parameters. The demonstrated anxiolytic- and antidepressant-like effects of SERAAK2 in animal models, which may involve its interaction with 5-HT_1A_ receptors, warrant further studies to confirm these activities and elucidate the underlying mechanisms.

## 1. Introduction

Central nervous system (CNS) disorders represent a major problem for healthcare systems, economies, and societies, particularly in high-income countries [1,2]. Their increasing prevalence, associated with aging populations, environmental factors, and lifestyle-associated risks, underscores the urgent need for improved diagnostic, therapeutic, and preventive strategies. CNS disorders are linked with a considerable morbidity, long-term disability, and substantial healthcare costs, thereby reducing individual well-being and societal productivity [3].

Schizophrenia, depression, anxiety disorders, and memory deficits are among the most frequently diagnosed mental disorders. They display a complex pathomechanism, involving a number of molecular targets, in particular belonging to aminergic G protein-coupled receptors (GPCRs). Among them, serotonin receptors are particularly important, as they regulate important physiological processes, such as mood, appetite, memory, and sexual behaviors. Consequently, they are targeted by numerous CNS agents like antipsychotics, anxiolytics, and antidepressants.

**Figure 1:**
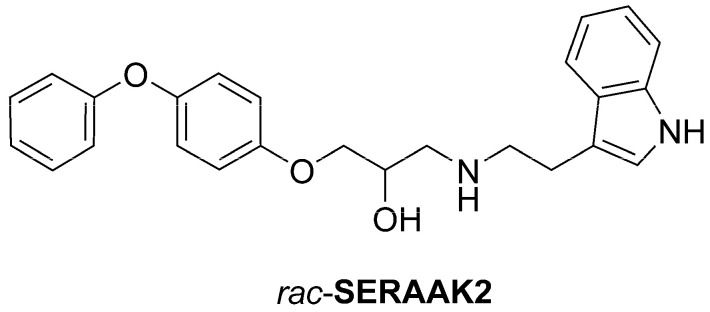
Compound SERAAK2.

As a part of our long-term research focus to discover potential CNS agents, we performed structure-based virtual screening aimed at searching for 5-HT_2A_ receptor ligands [4]. Six virtual hits were identified and validated in vitro. Two compounds, i.e., previously published SERAAK1 [5] and presented here SERAAK2 (Figure 1), underwent detailed in silico, in vitro, and in vivo studies.

SERAAK2 has a submicromolar affinity to the serotonin 5-HT_2A_ (*K_i_*: 422 ± 74 nM) and 5-HT_1A_ receptors (*K_i_*: 153 ± 0.4 nM), a moderate affinity to the 5-HT_7_ receptor (*K_i_*: 914 ± 25 nM), and no affinity to the dopamine D_2_ receptor (percent of inhibition at 10 μM 23 ± 6%) [4]. To bridge the gap between in vitro studies and more advanced preclinical research, we performed molecular docking and molecular dynamics simulations to study the ligand–receptor interactions, experimentally determine the ADMET parameters of SERAAK2, and carry out a behavioral assessment of this compound. Here, we report the pharmacological characterization of SERAAK2 as a novel serotonin receptor ligand investigated through a combination of in silico, in vitro, and in vivo approaches.

## 2. Results

### 2.1. In Silico Studies

#### 2.1.1. Molecular Docking

During virtual screening of the Enamine database, which led to the discovery of SERAAK2 [4], all the possible stereoisomers of the screened compounds were investigated. The S-enantiomer of SERAAK2 achieved the highest docking score and was, therefore, selected for further in silico studies. The compound was bought from Enamine as a racemate and, as such, was used in all experimental evaluations.

**Figure 2:**
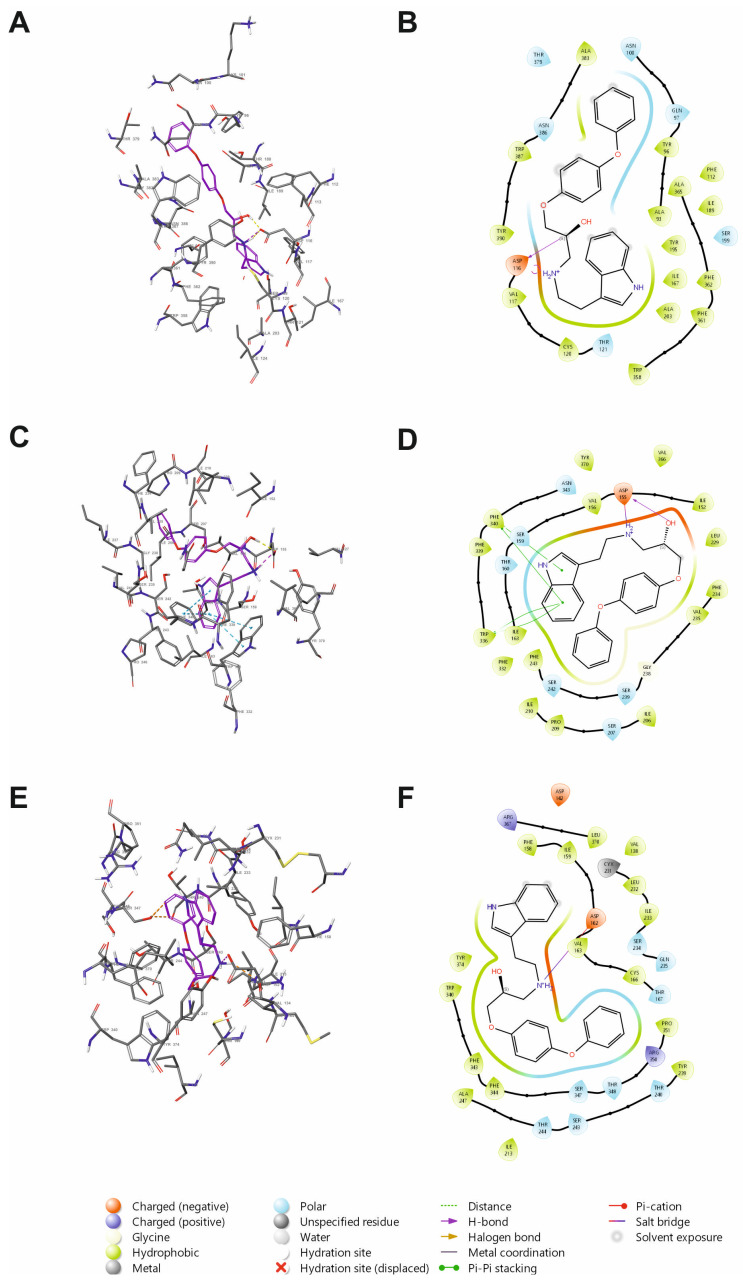
Docking poses of SERAAK2 within the orthosteric binding sites of the 5-HT_1A_ (**A**,**B**), 5-HT_2A_ (**C**,**D**), and 5-HT_7_ receptors (**E**,**F**). Panels (**A**,**C**,**E**) show the 3D arrangement of SERAAK2 in the receptor binding pocket. The ligand is displayed as sticks with magenta carbon atoms, and receptor residues are shown as sticks with gray carbon atoms. Non-polar hydrogens are omitted for clarity. Key ligand–receptor interactions are indicated by dashed lines (hydrogen bonds—yellow; salt bridges—magenta; π–π stacking—blue). Panels (**B**,**D**,**F**) present the corresponding 2D interaction maps.

SERAAK2 was docked with Glide (Schrödinger, v. 2024.2) to the orthosteric binding sites of the serotonin 5-HT_1A_, 5-HT_2A_, and 5-HT_7_ receptors in order to study ligand–receptor interactions at the molecular level. For the 5-HT_1A_ receptor, the cryo-EM structure bound to a partial agonist aripiprazole was used (PDB ID: 7E2Z) [6]; for the 5-HT_2A_ receptor, the X-ray structure in complex with an antagonist risperidone (PDB ID: 6A93) was selected [7]; and for the 5-HT_7_ receptor, the cryo-EM structure in complex with an agonist 5-CT (PDB ID: 7XTC) [8]. The molecular docking results are presented in Figure 2.

Molecular docking simulations were successfully validated by a re-docking experiment of co-crystallized reference ligands. The RMSD between the X-ray and the docked pose measured on heavy atoms only was 1.4202 Å for the 5-HT_1A_ receptor ligand, 2.0290 Å for the 5-HT_2A_ receptor ligand, and 0.8996 Å for the 5-HT_7_ receptor ligand. The overlay of X-ray and docking poses is shown in Figure S1 in the Supporting Information.

Across the receptors, the salt bridge interaction between the protonated amine of SERAAK2 and the conserved Asp3.32 from the third transmembrane helix of the receptor (Asp116, Asp155, and Asp162 in the 5-HT_1A_, 5-HT_2A_, and 5-HT_7_ receptor, respectively) was maintained, consistent with data for aminergic GPCR pharmacophores. This finding primarily explains the affinity rather than the efficacy of the compound toward the 5-HT receptors [9,10].

In the 5-HT_1A_ receptor, the SERAAK2 hydroxylic group makes an additional hydrogen bond with Asp3.32 (Figure 2A,B). In addition, here, SERAAK2 adopts a fully extended conformation with the indole moiety penetrating deep into the receptor TM6 hydrophobic cage (formed by Trp6.48 [Trp358], Phe6.51 [Phe361], and Phe6.52 [Phe362]), whereas the terminal phenoxy is directed to the receptor extracellular vestibule. Benchmarking against the 5-HT_1A_–aripiprazole complex reproduces the engagement of this aromatic cage. This extended pose may partially explain the highest affinity of SERAAK2 to the 5-HT_1A_ receptor. Such a conformation was not observed in other studied receptor–ligand complexes.

In contrast, in the 5-HT_2A_ receptor, SERAAK2 adopts a bent conformation, following other trends identified in the SERAAK2-5HT_1A_ receptor complex, i.e., additional interaction between the ligand hydroxylic group and Asp3.32 and orientation of the indole moiety towards the hydrophobic cage (Trp6.48 [Trp336], Phe6.51 [Phe339], and Phe6.52 [Phe340]); see Figure 2C,D. In addition, SERAAK2 forms π-π stacking interactions with Trp6.48 and Phe6.62.

In 5-HT_7,_ SERAAK2 also adopts a bent pose with both phenoxy and indole moieties oriented toward the extracellular part of the receptor, and there are no consistent secondary hydrogen bonds beyond Asp3.32 (Figure 2E,F). The absence of additional stabilizing contacts helps to rationalize the weakest binding among the three receptors.

Overall, the docking results confirm that SERAAK2 adopts a receptor-dependent pose preference—extended in the 5HT_1A_ receptor versus bent in the binding pockets of 5-HT_2A_/5-HT_7_. Differences in secondary interactions reflect variability in the experimental affinity toward these molecular targets. Efficacy (antagonism vs. agonism) cannot be inferred from docking alone and typically requires more dynamic evidence.

#### 2.1.2. Molecular Dynamics Simulations

**Figure 3:**
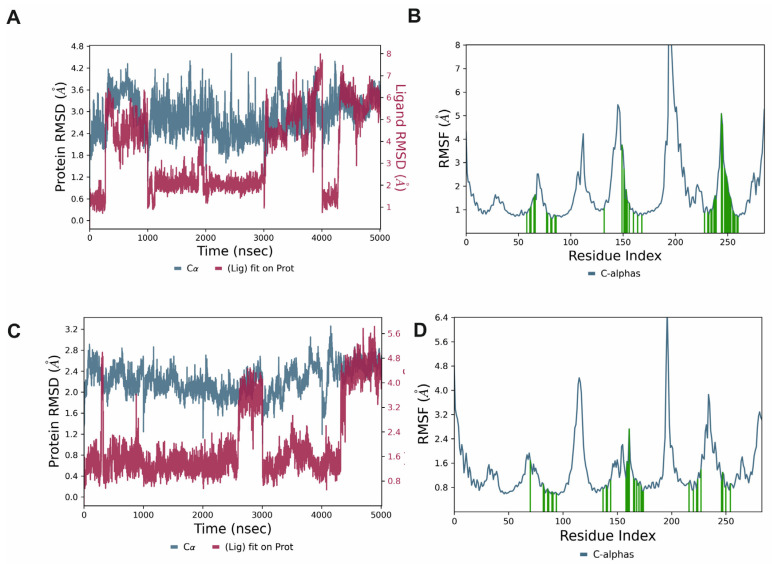
Ligand and protein RMSD (**A**,**C**) and protein RMSF (**B**,**D**) during molecular dynamics simulations for SERAAK2-5-HT_1A_ (**A**,**B**) and SERAAK2-5-HT_2A_ (**C**,**D**) receptor complexes.

In order to examine the dynamic nature of the protein–ligand complex formation, five SERAAK2 replicas with the 5-HT_1A_ and 5-HT_2A_ receptors were subjected to molecular dynamics simulations with Desmond (Schrödinger, v. 2024.2). Graphical depictions of the ligand and protein root mean square deviation (RMSD) changes are depicted in Figure 3A,C. For the majority of the simulations, SERAAK2 was stable in both receptors.

The residue root mean square fluctuation (RMSF) analysis (Figure 3B,D) shows the expected pattern for proteins belonging to class A of GPCRs. In particular, loops and extracellular regions exhibit greater mobility, whereas transmembrane helical segments remained comparatively rigid, consistent with well-packed GPCR cores.

**Figure 4:**
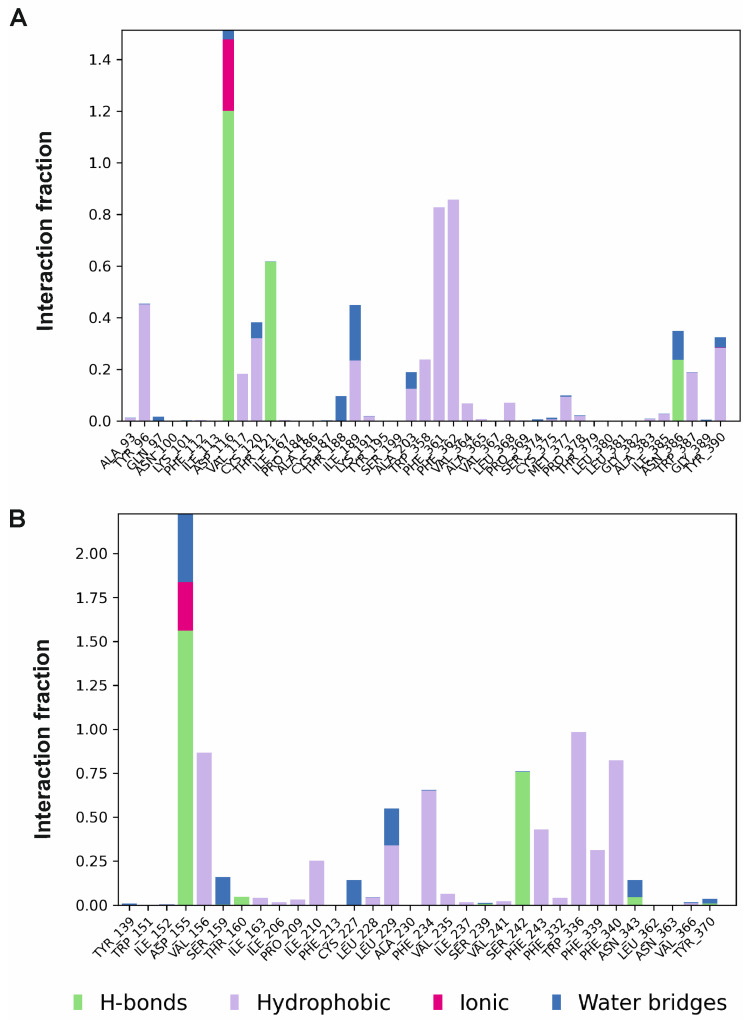
Normalized histograms of ligand–receptor contacts in the simulated complexes of SERAAK2 with the 5-HT1A (**A**) and 5-HT2A (**B**) receptors. The stacked bar plots represent contact frequencies averaged over the simulation trajectory. Values reaching 1.0 may occur when individual protein residues form multiple contacts of the same subtype as the ligand.

SERAAK2 adopted an extended conformation in the 5-HT_1A_ receptor stabilized by several interactions shown in the histogram in Figure 4A. SERAAK2, simulated inside the binding pocket of the 5-HT_2A_ receptor, kept a bent conformation, mirroring the docking results.

Contact occupancy analysis over the trajectories revealed that for the 5-HT_1A_ receptor complex, the main anchoring point of SERAAK2 is Asp3.32 (Asp116 in 5-HT_1A_ and Asp155 in 5-HT_2A_), which is reflected by the tallest bar on the histograms depicted in Figure 4.

In 5-HT_1A_, SERAAK2 further interacted with residues of the aromatic triad of TM6-Trp6.48 (Trp358), Phe6.51 (Phe361), and Phe6.52 (Phe362) via hydrophobic interactions and formed hydrogen bonds to Thr3.37 (Thr121) and Asn7.38 (Asn386) (Figure 4A).

In the case of the 5-HT_2A_ receptor complex, a significant hydrogen bond was identified with Ser5.46 (Ser242), which is in accordance with lower SERAAK2 affinity to this receptor. In addition, π- contacts with Trp6.48 (Trp336)—an important “toggle-switch” residue—were also reported.

Overall, the MD simulation results highlight the importance of conserved residues in ligand binding in 5-HT_1A_ and 5-HT_2A_ receptor binding pockets. Additional subtype-specific contacts provide a structural rationale for observed selectivity. However, at the same time, this reliance on conserved motifs highlights a potential risk of cross-selectivity with other aminergic GPCRs and should be addressed by other computational and experimental analyses.

**Figure 5:**
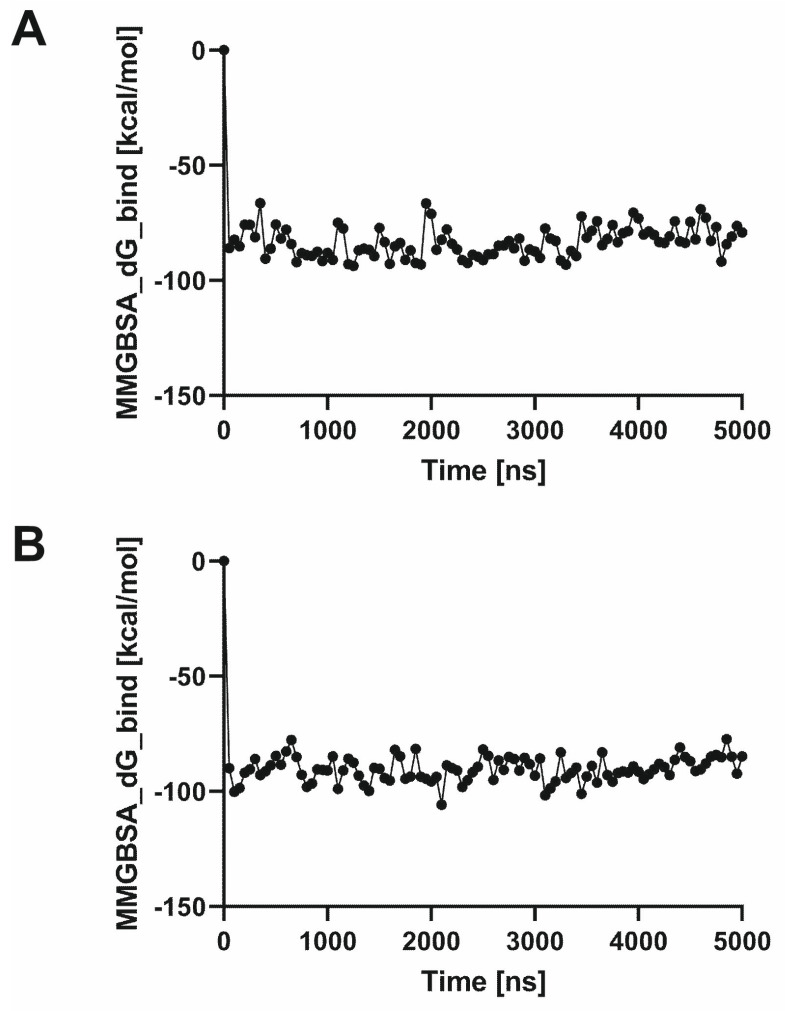
MM-GBSA dG bind over simulation time for SERAAK2-5HT_1A_ (**A**) and SERAAK2-5-HT_2A_ (**B**).

Molecular dynamics simulations were followed by molecular mechanics with generalized Born and surface area solvation (MM-GBSA) calculations to reflect SERAAK2 binding affinity to the studied receptors (Figure 5), further supporting experimental data.

**Figure 6:**
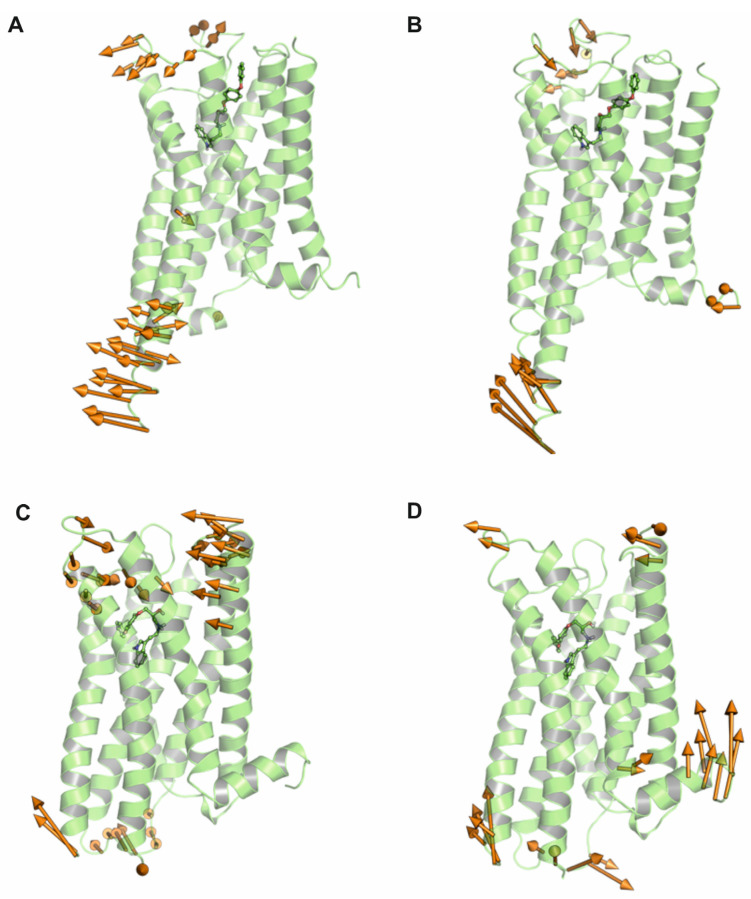
Porcupine plots illustrating protein–ligand complexes following molecular dynamics simulations. (**A**,**B**) show PC1 and PC2 for the SERAAK2-5-HT_1A_ receptor complex. (**C**,**D**) show B–PC1 and PC2 for the SERAAK2-5-HT_2A_ receptor complex.

In the final stage of the workflow, principal component analysis (PCA) was applied to trajectories to gain deeper insights into the complex’s motion. This method allowed the identification of the key components driving protein dynamics. The corresponding results are presented in Figure 6. Examination of porcupine plots showed that these movements were mainly concentrated in loop regions and neighboring helices, underscoring the importance of conformational flexibility in these areas for the protein’s spatial dynamics.

### 2.2. ADMET Evaluation of SERAAK2

The assessment of ADMET (Absorption, Distribution, Metabolism, Excretion, and Toxicity) properties is fundamental in drug discovery, as it determines a compound’s bioavailability, safety, and overall therapeutic potential. A pharmacologically active molecule is ineffective if it cannot reach its target or possesses unacceptable toxicity. Therefore, early ADMET profiling is crucial for drug development by eliminating problematic candidates early and guiding the optimization of lead compounds, thereby increasing the likelihood of clinical success.

Consequently, the next stage of the research was designed to characterize SERAAK2 ADMET parameters. This involved determining its potential to inhibit the cytochrome P450 enzymes 3A4 and 2D6, evaluating its cytotoxic effects on HepG2 and SH-SY5Y cell models, and measuring its passive diffusion across membranes via the PAMPA model.

#### 2.2.1. Cytochrome P450 Enzymatic Activity

The effect of SERAAK2 on CYP3A4 activity was assessed using a luminescent luciferase-based assay, which enables quantitative evaluation of substrate metabolism by cytochrome P450 enzymes. This method relies on measuring the light intensity produced by a luciferase-catalyzed reaction, in which the luminescent signal is directly proportional to enzymatic activity.

The inhibitory activity was determined by measuring the reduction in luminescence, expressed as a percentage relative to control benchmarks for full inhibition (ketoconazole, IC_50_: 156 nM) and baseline activity (0.2% DMSO). The data, presented in Table 1, reveal that SERAAK2 at the tested concentration did not inhibit the activity of CYP3A4 (0 ± 2%), effectively ruling out a meaningful pharmacological interaction with CYP3A4 and indicating a favorable profile regarding metabolism-based interactions.

The inhibitory potential of the compound against the CYP2D6 isoform was evaluated employing a methodology analogous to the CYP3A4 assay, albeit with a different set of reference compounds. In this configuration, complete enzyme inhibition was defined by treatment with 10 µM quinidine, while the baseline for 100% enzymatic activity was established using a 0.2% DMSO solution in phosphate buffer. The quantitative outcomes of this investigation are compiled and displayed in Table 2.

**Figure 7:**
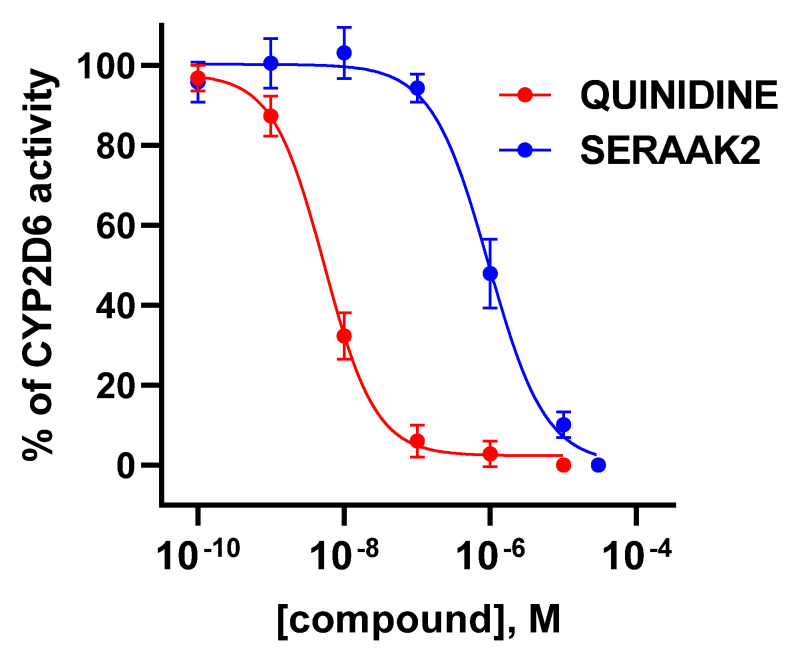
Dose–response profiles for CYP2D6 inhibition by quinidine (reference) and SERAAK2 (test compound).

At 10 µM, SERAAK2 inhibited the activity of the 2D6 isoform by 90%. Further dose–response analysis revealed that SERAAK2 inhibited CYP2D6 in a about one-micromolar range (Figure 7). To further examine this property, SERAAK2 was forwarded to an evaluation in a wider range of concentrations, yielding an IC_50_ of 1 µM. Taken together with the lack of activity against the 3A4 isoform, these findings indicate significant selectivity towards both isoforms.

#### 2.2.2. Cell Cytotoxicity

**Figure 8:**
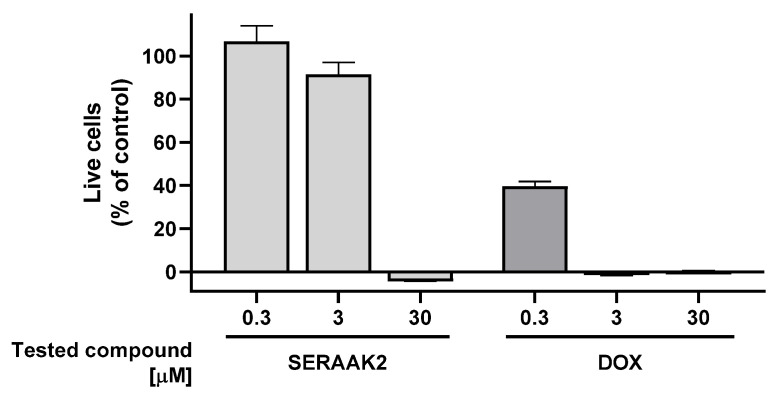
Cytotoxicity profile in HepG2 cells. Cell viability was measured after compound exposure; averaged from two separate experiments (*n* = 3 per experiment). Doxorubicin (DOX) served as a reference compound.

**Figure 9:**
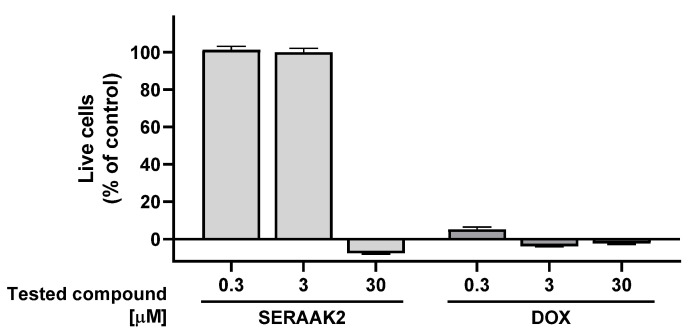
Cytotoxicity assessment in SH-SY5Y cells. Viability data are shown as a percentage of viable cells relative to control from two separate experiments (*n* = 3 per experiment). Doxorubicin (DOX) served as a reference compound.

The cytotoxic potential of SERAAK2 was evaluated in two human-derived cell lines chosen for their physiological relevance: HepG2 (hepatocellular carcinoma) and SH-SY5Y (neuroblastoma). Cell viability was quantified using the MTS assay, and the results are presented in Figure 8 and Figure 9. At the highest concentration tested, SERAAK2 demonstrated potent anti-proliferative effects in both models. The compound completely suppressed growth in SH-SY5Y cells (108 ± 1% inhibition) and showed similarly strong cytotoxicity against HepG2 cells (104 ± 1% inhibition), indicating broad cytotoxic activity across different cell types. It is particularly noteworthy that the use of the SERAAK2 compound at a concentration of 30 µM reduces the number of viable cells below the level of the positive control (doxorubicin) at the same concentration (Table 3). Doxorubicin is an anticancer drug that primarily inhibits cell proliferation rather than exerting direct cytotoxic effects. Under the experimental conditions used, after 72 h of incubation, a fraction of doxorubicin-treated cells remains metabolically active and generates a low signal in the MTS assay. The enhanced signal reduction observed in the MTS assay for the tested compound, compared to doxorubicin, indicates strong cytotoxic activity leading to necrosis of the entire cell population.

#### 2.2.3. Membrane Permeability

The Parallel Artificial Membrane Permeability Assay (PAMPA) is an in vitro method for assessing a compound’s passive diffusion across lipid membranes. By measuring the transfer from a donor to an acceptor compartment, the assay provides a permeability coefficient (P_e_) that predicts potential absorption or membrane penetration. PAMPA is widely used in early drug development due to its speed, simplicity, and cost-effectiveness in screening compounds for favorable pharmacokinetic properties.

In this study, PAMPA was employed to evaluate the passive diffusion of SERAAK2 through a lipid-infused artificial membrane [11]. The results, summarized in Table 4, are expressed as the permeability coefficient (P_e_), calculated according to earlier reported equations [12]. Caffeine, known for high permeability, and sulpiride, with low permeability, were included as reference compounds to contextualize the measurements.

Overall, for SERAAK2, an indole derivative containing a hydroxy group, the P_e_ coefficient value was slightly lower than that indicated by the manufacturer of the PAMPA test as the threshold value allowing us to classify this compound as well penetrating biological membranes (1.10 × 10^−6^ cm/s < 1.5 × 10^−6^ cm/s).

### 2.3. Behavioral Studies

#### 2.3.1. Spontaneous Locomotor Activity

**Figure 10:**
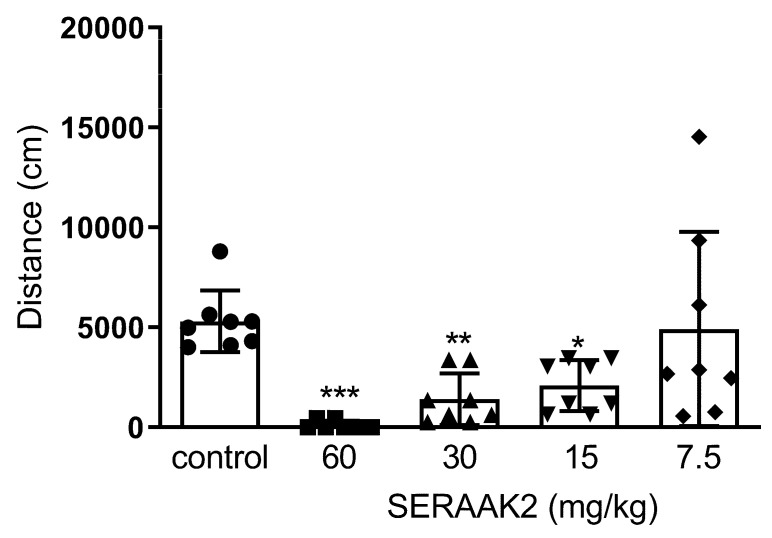
The effects of SERAAK2 (at doses of 60, 30, 15, and 7.5 mg/kg, i.p.) on spontaneous locomotor activity of mice. The tested compound was injected 60 min prior to testing, and activity was recorded over 20 min. Data are expressed as mean ± SEM from a single independent experiment. *** *p* < 0.001, ** *p* < 0.01, * *p* < 0.05 vs. control (Dunnett’s test).

One-way ANOVA revealed significant alterations in locomotor activity of mice 20 min after administration of the compound SERAAK2 at doses of 60, 30, 15, and 7.5 mg/kg (F(4,39) = 6.955; *p* < 0.001). Dunnett’s post hoc analysis confirmed a statistically significant reduction in locomotor activity of mice after SERAAK2 administration at doses of 60 mg/kg (*p* < 0.001), 30 mg/kg (*p* < 0.01), and 15 mg/kg (*p* < 0.05) following 20 min of observation (Figure 10).

#### 2.3.2. Motor Coordination

**Figure 11:**
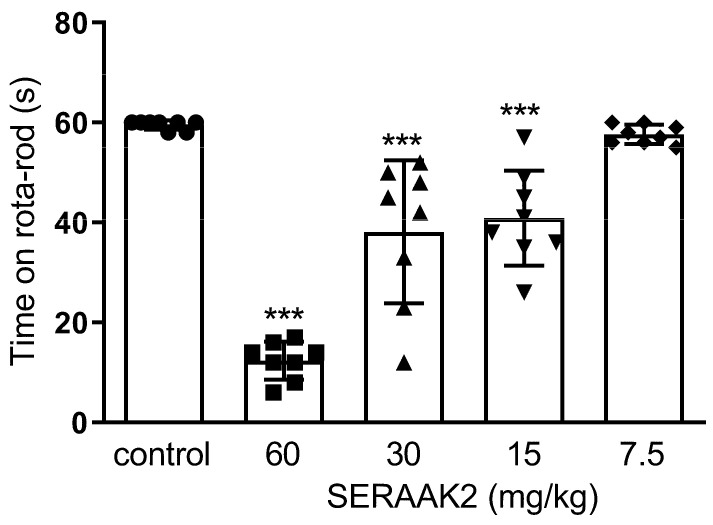
The effects of SERAAK2 (at doses of 60, 30, 15, and 7.5 mg/kg, i.p.) on motor coordination in mice in the rotarod test. The compound was injected 60 min prior to testing. Data are expressed as mean ± SEM from a single independent experiment. *** *p* < 0.001 vs. control (Dunnett’s test).

**Figure 12:**
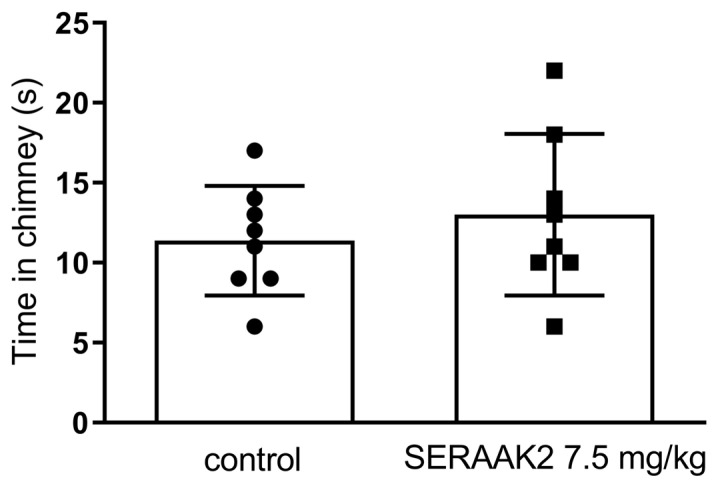
The effects of SERAAK2 (at a dose of 7.5 mg/kg, i.p.) on motor coordination in mice in the chimney test. The compound was injected 60 min prior to testing. Data are expressed as mean ± SEM from a single independent experiment.

One-way ANOVA revealed significant disturbances in motor coordination of mice after administration of the compound SERAAK2 at doses of 60, 30, and 15 mg/kg in the rotarod test (Figure 11). Mice that received SERAAK2 at a dose of 7.5 mg/kg were held on a rotating rod for about 60 s (Figure 11) and left the chimney in a time similar to that of the control group (about 13 s; Figure 12).

#### 2.3.3. Effect of Acute Administration of SERAAK2 (7.5 mg/kg) on Elevated Plus Maze (EPM) Performance in Mice

**Figure 13:**
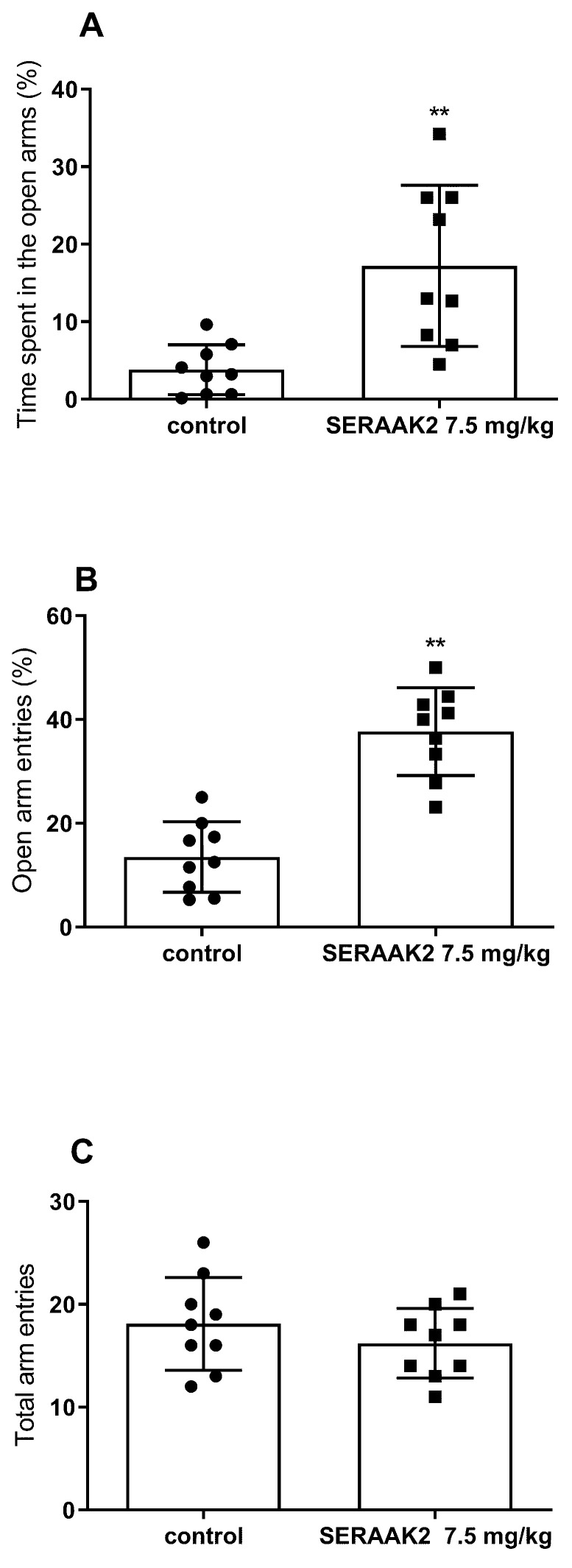
The effects of SERAAK2 (at a dose of 7.5 mg/kg, i.p.) on elevated plus maze performance in mice—percentage of time spent in open arms (**A**), percentage of the open arm entries (**B**), and total arm entries (**C**). The tested compound was injected 60 min prior to testing. Data are expressed as mean ± SEM from a single independent experiment. ** *p* < 0.01, vs. control (Student’s *t*-test).

Statistical analysis using Student’s *t*-test showed that SERAAK2 (7.5 mg/kg) produced an anxiolytic-like effect, as evidenced by a significant increase in both the percentage of time spent in the open arms (*p* < 0.05; Figure 13A) and the percentage of open arm entries (*p* < 0.01; Figure 13B). Importantly, the total number of entries into both open and closed arms remained unchanged, indicating no impact on general locomotor activity (Figure 13C).

#### 2.3.4. Effect of Acute Administration of SERAAK2 (7.5 mg/kg) on the Total Duration of Immobility in the Forced Swim Test (FST) in Mice

**Figure 14:**
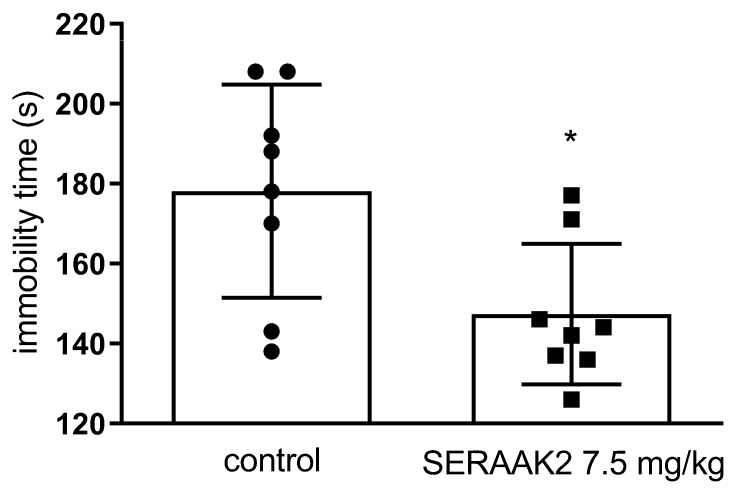
The effects of SERAAK2 (at a dose of 7.5 mg/kg, i.p.) on the immobility time in the forced swim test in mice. The tested compound was injected 60 min before testing. Data are expressed as mean ± SEM from a single independent experiment. * *p* < 0.05 vs. control (Student’s *t*-test).

Statistical analysis using Student’s *t*-test showed that SERAAK2 (7.5 mg/kg) produced an antidepressant-like effect, as evidenced by a significant decrease in immobility time (*p* = 0.001; Figure 14).

#### 2.3.5. Effect of Acute Administration of SERAAK2 (7.5 mg/kg) on Memory Consolidation in the Passive Avoidance (PA) Test in Mice

The effect of SERAAK2 (7.5 mg/kg) on memory consolidation was evaluated during the retention phase of the passive avoidance test. Statistical analysis using Student’s *t*-test indicated that administration of the tested compound did not significantly change the latency index (IL) values vs. the vehicle-treated control group.

#### 2.3.6. Amphetamine-Induced Hyperactivity in Mice

**Figure 15:**
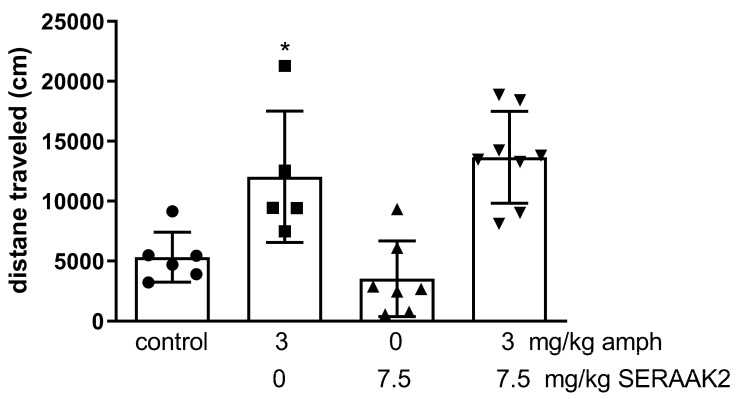
The effects of SERAAK2 (at a dose of 7.5 mg/kg, i.p.) on amphetamine-induced hyperactivity in mice. The compound was injected 60 min prior to testing, followed by amphetamine (3 mg/kg) 30 min later. Data are expressed as mean ± SEM from a single independent experiment. * *p* < 0.05 vs. control (Dunnett’s test).

Statistical analysis revealed significant changes in the locomotor activity after SERAAK2 (7.5 mg/kg) treatment in combination with amphetamine (two-way ANOVA: pretreatment [F(1,24) = 16.92, *p* < 0.0001], treatment [F(1,24) = 0.13; *p* = 0.07243], and interaction effect between pretreatment and treatment [F(1,24) = 0.39, *p* = 0.05382]). The administration of amphetamine alone significantly increased the locomotor activity of mice compared to the saline-treated group (*p* < 0.05). However, the co-administration of SERAAK2 did not significantly modify this hyperactivity in comparison to the amphetamine-treated group (Dunnett’s post hoc test; Figure 15).

## 3. Discussion

Compound SERAAK2 was identified in a structure-based virtual screening [4]. Here, we present further evaluation of this compound as a potential CNS agent.

The performed molecular modeling studies confirmed that the binding mode of SERAAK2 to the studied receptors is typical for aminergic GPCR ligands with the conserved Asp3.32 as the main anchoring point, as reported earlier for related chemotypes [5,13,14,15], including SERAAK1. The indole moiety of SERAAK2 is directed to the aromatic cage of the receptors formed, i.a., by Trp6.48, Phe6.51, and Phe6.52, which is in accordance with the previous studies [5,13,14,15], including the earlier reported results for SERAAK1. Multi-microsecond molecular dynamics simulations supported the molecular docking results, confirming pose persistence and orthosteric stability. Moreover, it proved that the main ligand–receptor contacts are maintained during the simulations. Moreover, PCA analysis revealed that the main motions concern flexible loops and helices termini, as earlier found for compound SERAAK1 [5]. Because SERAAK’s binding highly relies on residues conserved across many GPCR sites, some degree of cross-reactivity is possible.

To evaluate SERAAK2 clinical potential, we conducted in vitro ADMET studies focusing on its interactions with cytochrome P450 enzymes, particularly the pharmacologically critical isoforms CYP3A4 and CYP2D6. These enzymes were prioritized due to their established roles in drug metabolism—CYP3A4 mediates the breakdown of approximately half of all marketed drugs, while CYP2D6 is essential for processing antidepressants, opioids, and cardiovascular agents. Notably, SERAAK2 showed a neutral effect on CYP3A4, distinguishing it from its structural analog SERAAK1 [5], which demonstrated potent CYP3A4 induction (3.5-fold increase). Regulatory agencies recommend CYP interaction screening during early drug development to assess potential drug–drug interactions.

Our analysis demonstrated that SERAAK2 exhibits a selective interaction with cytochrome P450 enzymes. The compound was found to inhibit the activity of CYP2D6 but did not affect CYP3A4. Since these assays were performed using purified recombinant enzymes, the results indicate a direct action of SERAAK2 on the catalytic function of these isoforms. The inhibition of CYP2D6 is likely due to SERAAK2 binding at or near the enzyme’s active site, either competitively by obstructing substrate entry or non-competitively by inducing a conformational change that impairs its activity. Importantly, these results describe only the interaction potential of SERAAK2 with individual CYP isoforms and do not provide information about its metabolic fate or in vivo stability, which would require dedicated pharmacokinetic studies. For reference, compounds with strong CYP2D6 or CYP3A4 inhibitory properties—such as paroxetine or amiodarone—are known to influence the plasma concentrations of co-administered drugs metabolized by these enzymes. Therefore, while CYP2D6 inhibition does not preclude the further development of this chemotype, it represents a developmental drawback that would require structural optimization to minimize the risk of clinically relevant drug–drug interactions.

We also evaluated the SERAAK2 impact on cell viability using two human-derived cell lines, HepG2 (hepatocellular carcinoma) and SH-SY5Y (neuroblastoma), which provide more clinically relevant toxicity predictions than non-human models. This dual-line approach allowed simultaneous assessment of potential hepatotoxicity and neurotoxicity.

The results showed no cytotoxic effects at low concentrations (0.3 µM). Moderate concentrations (3 µM) induced slight cytotoxicity in HepG2 cells, reducing viability to 85% of the control. However, at 30 µM, SERAAK2 completely inhibited cell growth through necrotic mechanisms, with stronger effects observed in hepatoma cells than neuroblastoma cells—potentially indicating liver-specific toxic metabolite formation.

In membrane permeability tests, SERAAK2 exhibited moderate artificial lipid bilayer penetration, falling slightly below the conventional threshold for highly permeable compounds but clearly higher than the poorly permeable reference sulpiride. While further optimization may be warranted, the overall ADMET profile of SERAAK2 appears promising and supports its continued development as a potential CNS-active drug candidate.

The performed ADMET profiling confirmed the improved developability of SERAAK2 when compared with our previously reported virtual hit SERAAK1 [5]. Although both molecules originated from the same structure-based virtual screening workflow, they represent distinct chemical scaffolds rather than a derivative analog series. This difference in chemotype is reflected in their markedly divergent in vitro ADMET properties. SERAAK1 showed no measurable passive permeability in the PAMPA assay (Pe < 0.010 × 10^−6^ cm/s), whereas SERAAK2 demonstrated substantially higher membrane diffusion. A similarly favorable trend was observed for cytochrome P450 interactions. SERAAK1 exhibited a strong and undesirable CYP3A4 activation together with potent CYP2D6 inhibition (IC_50_ = 720 nM). In contrast, SERAAK2 showed no inhibitory or stimulatory effect on CYP3A4, and although it remained a CYP2D6 inhibitor, its activity was noticeably weaker. Taken together, these experimentally observed differences clearly demonstrate that SERAAK2 surpasses SERAAK1 in several key ADMET dimensions. Consequently, SERAAK2 emerges as a more promising starting point for further development within this newly identified class of serotonin receptor ligands.

The functional profiling of SERAAK2 continued with in vivo experiments. An initial series of experiments assessed locomotor activity and motor coordination, which are widely recognized as fundamental benchmarks for screening the central activity of novel substances [16]. At the higher doses administered (60, 30, and 15 mg/kg), SERAAK2 significantly decreased the spontaneous locomotor activity of the animals and impaired motor coordination in the rotarod and chimney tests, whereas the lowest dose tested (7.5 mg/kg) was inactive in the locomotor and coordination tests. This inactive dose (7.5 mg/kg) was subsequently used to assess animal behavior in the other tests.

As severe mental health conditions that disrupt daily life and incur substantial public health costs, anxiety and stress-related disorders are a major concern. To investigate the anxiolytic properties of SERAAK2, we employed the elevated plus maze test (EPM), a well-established model founded on the innate avoidance behavior rodents exhibit towards open areas [17]. SERAAK2 at a dose of 7.5 mg/kg increased entries and time spent at the open arms without affecting overall activity, suggesting a mechanism specifically reducing anxiety-related behavior. Activity in this assay is demonstrated, among others, by buspirone, a non-benzodiazepine anxiolytic used in the treatment of anxiety disorders. Buspirone acts as a partial agonist at 5-HT_1A_ receptors and modulates serotonergic transmission within limbic brain regions, which are strongly implicated in the pathophysiology of anxiety [18]. It is accepted that buspirone’s anxiolytic effects are mainly mediated by 5-HT_1A_ receptors [19]. Buspirone represents a well-tolerated alternative to benzodiazepines, which are associated with sedation and may lead to tolerance and dependence with prolonged use. Given its predominant 5-HT_1A_ receptor profile, the anxiolytic-like effects of SERAAK2 may, similarly to buspirone, arise from its interaction with 5-HT_1A_ receptors [18].

The antidepressant potential of SERAAK2 was evaluated using the forced swim test (FST), a well-established and rapid screening method in experimental pharmacology for assessing antidepressant activity substances [20]. In this model, depressive-like behavior is reflected by immobility, where the animal remains floating with only minimal movements. Compounds with antidepressant properties reduce immobility time, whereas those with depressogenic effects increase it [21]. Selective serotonin reuptake inhibitors (SSRIs) serve as a reference class of antidepressants active in this test; they inhibit the serotonin transporter (SERT), thereby reducing serotonin reuptake from the synaptic cleft. Administration of SERAAK2 at a dose of 7.5 mg/kg reduced the immobility time in mice, indicating antidepressant-like activity.

Mental disorders such as depression are frequently accompanied by cognitive impairments, including deficits in learning and memory [22]. Based on this rationale, we evaluated the potential cognitive effects of SERAAK2 using the passive avoidance task to assess long-term memory. Rodents naturally prefer darker environments. During the acquisition phase of the task, animals receive mild electric foot shocks upon entering a dark compartment. Following this training, mice typically avoid or delay re-entering the dark chamber during the retention trial, indicating memory of the aversive stimulus. The absence of an effect of SERAAK2 in this test suggests that the compound does not influence the cognitive processes evaluated by this paradigm, particularly memory consolidation.

SERAAK2 did not affect amphetamine-induced hyperactivity in mice, which can be linked to its lack of affinity to the D_2_ receptor.

The observed anxiolytic- and antidepressant-like effects of SERAAK2 in mice, combined with its favorable ADMET profile, support further development of this compound and evaluation of its activities in more advanced animal models to confirm and elucidate the underlying mechanisms.

Comparison of the behavioral profiles of SERAAK1 and SERAAK2 reveals some differences in their effects on motor coordination and the mechanisms underlying their anxiolytic and antidepressant effects. Both compounds demonstrated activity in classical behavioral models, increasing open-arm exploration in the elevated plus maze and reducing immobility time in the forced swim test, suggesting their anxiolytic and antidepressant potential. However, SERAAK1, administered at doses of 30–60 mg/kg, did not affect spontaneous locomotor activity or motor coordination, whereas SERAAK2, at higher doses (15–60 mg/kg), significantly decreased activity and impaired coordination, suggesting sedative or toxic effects at higher doses. Moreover, SERAAK1 reduced amphetamine-induced hyperactivity, whereas SERAAK2 did not, consistent with its lack of affinity for dopamine receptors. From a receptor mechanism perspective, these differences can be interpreted in the context of the affinity profile of both molecules. SERAAK1 acts as a 5-HT_2_A antagonist, which may explain its ability to reduce amphetamine-induced hyperactivity and its effects on mood, analogous to atypical antipsychotics and some antidepressants. SERAAK2, on the other hand, exhibits higher affinity for 5-HT_1A_ and moderate affinity for 5-HT_2A_, likely exerting its anxiolytic effects through a mechanism similar to that of buspirone, a 5-HT_1A_ partial agonist, modulating serotonergic transmission in limbic structures. The observed antidepressant effect of SERAAK2 in the FST may also be due to 5-HT_1A_ activation, whereas the lack of effect on memory in the passive avoidance test suggests limited effects on cognitive processes. In summary, both compounds demonstrate a favorable profile in reducing symptoms of anxiety and depression, which is summarized in Table 5.

## 4. Materials and Methods

### 4.1. In Silico Studies

Compound SERAAK2 was modeled using LigPrep from the Schrödinger suite of software v. 2024.2. To address the compound protonation states, the Epik module of the Schrödinger suite was used. The following protein models were taken from the Protein Data Bank: the 5-HT_1A_ receptor cryo-EM structure in a complex with a partial agonist aripiprazole (PDB ID: 7E2Z) [6], the 5-HT_2A_ receptor with its X-ray structure in a complex with an antagonist risperidone (PDB ID: 6A93) [7], and the 5-HT_7_ receptor cryo-EM structure in a complex with a 5-CT agonist (PDB ID: 7XTC) [8]. Proteins were prepared using Protein Preparation Wizard from the Schrödinger suite of software. In particular, lacking extracellular loops were completed, residue protonation states were assigned, hydrogen atoms were added, and the models were subjected to restrained minimization.

SERAAK2 was docked to the prepared protein models using the standard precision (SP) method of Glide [23] from the Schrödinger suite. Grid files were generated based on co-crystallized ligands at default settings, enabling the flexibility of hydroxyl groups of the respective residues in the binding site. A total of 50 poses were obtained in each case, and the best poses were selected based on the Glide score and visual inspection.

In order to validate molecular docking simulations, a re-docking experiment was performed. Co-crystallized ligands were extracted from respective protein–ligand complexes, prepared with LigPrep and Epik, and docked following the docking protocol for SERAAK2. In each case, the first pose of the reference ligand was selected. The RMSD between the X-ray pose and the docked pose was measured on the heavy atoms using Discovery Studio Visualizer v. 17.1.0.16143.

SERAAK2 complexes with 5-HT_1A_ and 5-HT_2A_ receptors were subjected to molecular dynamics simulations using Desmond [24] from the Schrödinger suite as previously reported [5]. The system was incorporated into a lipid bilayer composed of POPC (1-palmitoyl-2-oleoyl-sn-glycero-3-phosphocholine), solvated with SPC (simple point charge) water molecules, and neutralized with the addition of NaCl to a final concentration of 0.15 M. Prior to production runs, the protein–ligand complexes underwent energy minimization and relaxation steps to eliminate steric clashes and achieve equilibrium conditions. Each simulation was executed for up to 1 μs per replica, with five independent replicas generated to improve sampling of the conformational landscape. Trajectory inspection was carried out with the Simulation Interaction Diagram utility in the Desmond suite. MM-GBSA [25] calculations were also performed over simulation time. To further probe conformational dynamics, including dominant motions, principal component analysis (PCA) was carried out using Schrödinger suite tools.

### 4.2. ADMET Studies

#### 4.2.1. Assessment of Cytochrome P450 Enzymatic Activity

The effect of SERAAK2 on the activity of the cytochrome P450 isoforms CYP3A4 and CYP2D6 was tested using commercial luminescence assays (Glo kits bought from Promega, Medison, WI, USA). The experiment was performed twice with triplicate samples, following the manufacturer’s guidelines and established procedures. A microplate reader measured the luminescence, and the results were compared to known inhibitors: ketoconazole for CYP3A4 and quinidine for CYP2D6 (luminescent output was detected using a Spark microplate reader from Tecan, Männedorf, Switzerland).

#### 4.2.2. In Vitro Cytotoxicity Assessment

An assessment of the cytotoxic potential of SERAAK2 was carried out utilizing the colorimetric MTS assay. This investigation was executed on both HepG2 and SH-SY5Y cell lines, adhering to an identical procedure. The cell lines, sourced from the American Type Culture Collection (Manassas, VA, USA), were cultured under standard conditions (5% CO_2_, 37 °C) in DMEM/F12 medium (#31330-038, ThermoFisher Scientific, Waltham, MA, USA). The medium was supplemented with 10% Fetal Bovine Serum (FBS, #A5256801, ThermoFisher Scientific) and a Penicillin–Streptomycin solution (#15140-122, ThermoFisher Scientific) until their use in the study. Cells within passage numbers 15 to 25 were plated in 96-well plates at a density of 5000 cells per well in 200 μL of medium and allowed to adhere during a 24 h incubation at 37 °C with 5% CO_2_. Following this, the medium was exchanged for fresh medium (120 μL), and serial dilutions of the test compound SERAAK2, which had been prepared in a mixture of DMSO and culture medium, were introduced (80 μL), achieving final concentrations of 30 μM, 3 μM, and 0.3 μM. The well-characterized cytostatic agent, doxorubicin, was employed as a reference compound. Control wells were established containing cells, medium, and 1% DMSO. The cells were exposed to the compounds for a duration of 72 h. Upon conclusion of the incubation period, the medium was removed, and an MTS/PBS reagent mixture in a 5:1 ratio was added to each well (120 μL). The plates were then subsequently returned to the 37 °C incubator, after which absorbance was recorded with a Spark microplate reader; this measurement occurred after 1 h for the HepG2 line and 3 h for the SH-SY5Y line. The ultimate cell viability percentage was determined by comparing results to the control wells, which represented 100% viability, and the wells treated with 10 μM doxorubicin, which were defined as 0% viability. This entire assay was performed in duplicate.

#### 4.2.3. Membrane Permeability Assessment

The membrane permeability assay was conducted employing the pre-coated PAMPA Plate System Gentest from Corning (Tewksbury, MA, USA), following the manufacturer’s provided instructions. SERAAK2, along with reference compounds, were prepared in PBS buffer at a pH of 7.4 to reach a final concentration of 100 µM, and these solutions were loaded onto the donor plate of the 96-well system. Following a 5 h incubation period, the precise amount of compound that had migrated from the donor wells to the acceptor wells, traversing the artificial phospholipid membrane, was quantified by UPLC-MS spectrometry using a Waters ACQUITY™ TQD system equipped with a TQ Detector (Waters, MLF, USA). The permeability coefficient (P_e_, expressed in cm/s) was subsequently derived by applying the calculation formula supplied by the manufacturer of the PAMPA system [12].

### 4.3. Behavioral Studies

#### 4.3.1. Animals

All experiments of the described studies took place in the Experimental Medicine Center, Medical University of Lublin, Poland, where the animals were also bred and maintained. The study used mice from the Swiss strain, which were 6 weeks old and whose weight ranged from 24 to 30 g, obtained from the in-house breeding colony of this facility. The animals were housed in groups of four to five per cage under standard laboratory conditions: ambient temperature maintained at 22 ± 1 °C, relative humidity between 50 and 60%, and a 12 h light/dark cycle with lights switched on at 8:00 a.m. Mice had unrestricted access to laboratory chow (LSM pellets, Agropol, Motycz, Poland) and tap water, except during short periods when they were removed for testing. The environment in which the animals stayed was enriched with cardboard tunnels and wooden blocks to improve the animals’ welfare. All experimental procedures complied with the European Directive 2010/63/EU for animal experiments and with the approval of the Local Ethics Committee for Animal Experimentation in Lublin (55/2022, issued on 4 April 2022). All behavioral testing was performed during the same period of the light phase (between 9:00 a.m. and 3:00 p.m.) under identical experimental conditions. The study comprised seven independent behavioral experiments performed on mice.

1. Spontaneous locomotor activity test—five groups (*n* = 8 per group): the control group received vehicle only, and four groups received the tested compound at doses of 7.5, 15, 30, or 60 mg/kg.
2. Rotarod test—same group design as above (five groups, *n* = 8 per group).
3. Chimney test,
4. FST
5. PA—two groups (*n* = 8 per group): control and the group receiving the compound at 7.5 mg/kg.
6. Elevated plus maze test—two groups (*n* = 9 per group): control and the group receiving the compound at 7.5 mg/kg.
7. Amphetamine-induced hyperactivity test—four groups (*n* = 8 per group): vehicle control, amphetamine only, tested compound at a dose of 7.5 mg/kg, and the combination of amphetamine and the tested compound.

Animals were randomly assigned to groups and treatment conditions before testing. The experimental unit was a single animal. Each mouse received an individual treatment according to group assignment and was analyzed independently. The same mice were used in the spontaneous locomotor activity test and rotarod test (40 animals total), as well as the amphetamine-induced hyperactivity test and chimney test (32 animals total). Total number of unique animals used was 122 (40 animals for the spontaneous locomotor/rotarod cohort + 32 animals for the amphetamine/chimney cohort + 16 animals for FST + 16 animals for PA + 18 animals for EPM = 122 animals.

No formal a priori power calculation was performed. Group sizes (generally *n* = 8 per group) were chosen based on prior published studies in the field and internal pilot data, balancing the need to detect biologically meaningful effects with ethical considerations to minimize animal use. The group size of *n* = 9 in the elevated plus maze was selected to allow for occasional data loss (e.g., dropouts or unusable recordings) observed in pilot experiments.

Animals were randomly assigned to control or treatment groups before the start of the experiments using simple randomization. The allocation sequence was manually generated by an independent investigator not involved in the behavioral testing to prevent allocation bias.

Several strategies were used to minimize potential confounders. Animals from different groups were tested in an alternating order to avoid time-of-day or order effects. The location of cages in the housing racks was regularly rotated to control for environmental factors such as light, temperature, and handling differences.

#### 4.3.2. Experiments

The primary outcome measure for hypothesis testing was the total distance traveled in the spontaneous locomotor activity test, as well as performance in the motor coordination tests, such as the rotarod and chimney test, which served as representative indicators of the compound’s CNS activity and potential neurotoxic effects and were used to estimate the appropriate sample size. Other behavioral tests provided complementary information, such as the time spent in open arms in the EPM test to assess anxiolytic-like activity, immobility time in the FST to evaluate antidepressant-like activity, and latency to enter the dark compartment in the PAe task to assess potential effects on long-term memory. The amphetamine-induced hyperactivity test was included as a confirmatory control to verify the absence of dopaminergic-mediated effects.

#### 4.3.3. Drugs

The investigated compound (SER2AAK2) was bought from Enamine (purity over 95%) and in all tests was administered intraperitoneally (i.p.), spread in a drop of Tween 80, and diluted with an aqueous solution of 0.5% methylcellulose (tylose) and injected 60 min before the tests in a volume of 10 mL/kg. Control groups received tylose injections of the same volume and via the same route of administration.

#### 4.3.4. Spontaneous Locomotor Activity and Amphetamine-Induced Hyperactivity

Locomotor activity of the mice was assessed using the Opto-Varimex-4 Auto-Track system (Columbus Instruments, OH, USA). Each mouse was placed individually in a testing cage for 25 min—5 min for acclimatization, followed by 20 min of activity recording. (6a) The total distance traveled (in centimeters) was measured. Cages were cleaned with 10% ethanol between sessions.

#### 4.3.5. Motor Coordination

The effects of the SER2AAK2 compound were also measured in the rotarod [26] and chimney [27] tests. In the rotarod test, mice were evaluated for their ability to maintain balance on a constantly rotating rod (18 rpm) for 1 min. The chimney test involved placing a mouse in a horizontal polymer tube (3 cm diameter, 25 cm length). Once the mouse reached the far end, the tube was positioned vertically, requiring the animal to climb backwards to exit. Right motor coordination was defined as exiting the chimney within 60 s. Prior to testing, mice underwent a 3-day training. Only those animals that were able to stay on the rotating rod or leave the chimney within the required time were included in the study.

#### 4.3.6. Elevated Plus Maze Test (EPM Test)

Anxiety behaviors were measured using the EPM test according to the Lister method [28]. The apparatus consisted of four black-painted arms arranged in a plus-shaped configuration with a central platform (5 × 5 cm). Two opposing arms were open (30 × 5 cm), while the other two were enclosed (30 × 5 × 15 cm). The maze was elevated 38.5 cm above the floor and illuminated with dim, diffuse red light. Experiments were conducted in a quiet, dark room. Each mouse was placed individually at the center of the maze, facing the open arm, and was observed for 5 min by an experimenter blinded to the treatment conditions. The following parameters were recorded: time spent in the open arms, number of entries into the open arms, and total number of entries into both types of arms.

#### 4.3.7. Forced Swim Test (FST, Porsolt’s Test) in Mice

The study was carried out using the test proposed by R. Porsolt [29]. The method is based on the observation of an animal forced to swim in a confined space from which there is no escape. After an initial period of vigorous attempts to get out, the animal finally gives up on escaping. The test consists of immersing the mouse individually in a cylindrical beaker (diameter 10 cm, height 25 cm) filled with water (at a temperature of 23–25 °C) at a height of 10 cm for 6 min. The immobility time was measured during the final 4 min of the test (between 2 and 6 min).

#### 4.3.8. Passive Avoidance Task

The passive avoidance (PA) task measures a long-term memory, as it was reported by Venault et al. [30]. The PA apparatus consists of a two-compartment acrylic box, one illuminated with fluorescent light (8 W) (10 cm × 13 cm × 15 cm) and another darkened (25 cm × 20 cm × 15 cm), connected by a guillotine door. Entrance of the animal to the darkened box was punished by an electric foot shock (0.15 mA for 2 s). The PA procedure was previously described [13]. In particular, on the first day of the experiment [i.e., training (pre-test)], mice were placed individually into the illuminated compartment and allowed to explore this box. After 30 s, the guillotine door was raised to allow the mice to enter the darkened compartment. When each mouse entered the darkened box, the guillotine door was closed, and an electric foot shock (0.15 mA) with a 2 s duration was delivered immediately to the animal via the grid floor. The latency time for entering the darkened compartment was recorded (TL1). If the mouse failed to enter the darkened box within 300 s, it was placed into this box (the door was closed), and an electric foot shock was delivered to the animal (TL1 value was recorded as 300 s). After 24 h, in the subsequent trial (retention), the same mouse was again placed individually in the illuminated compartment of the PA apparatus. After an adaptation period (30 s), the door between the two compartments was raised, and the mouse had time (300 s) to re-enter the dark compartment (TL2). No foot shock was applied in this trial. If the animal did not enter the dark compartment within 300 s, the test was stopped, and TL2 was recorded as 300 s. The experimental procedure involved examination of the memory consolidation when animals received the tested compound SERAAK2 (30 mg/kg; i.p.) or vehicle (control group) after the pre-test. The results were expressed as the latency index (LI), calculated by the following formula:

$$\frac{TL1-TL2}{TL1}$$

where *TL*1 is the time needed to move to the dark compartment during a training session and *TL*2 is the time needed to re-enter the dark compartment, measured 24 h after *TL*1 (retention).

#### 4.3.9. Statistical Analysis

Statistical analyses were performed using one-way analysis of variance (ANOVA), followed by Dunnett’s post hoc test. When appropriate, Student’s *t*-test was also applied. Data are presented as mean  ±  standard error of the mean (SEM). A *p*-value of less than  0.05 was considered statistically significant. All analyses and figures were created by GraphPad Prism version 5.00 for Windows (GraphPad Software, San Diego, CA, USA; www.graphpad.com).

No formal inclusion or exclusion criteria were established a priori. All animals that were allocated to the experimental groups and completed the procedures were included in the analysis. Data points were only excluded in cases of technical issues (e.g., equipment malfunction or animal escape during behavioural testing), which occurred rarely.

No animals or experimental units were excluded from the analysis due to treatment-related reasons. Occasional exclusions (if any) were due solely to technical problems during behavioral testing (e.g., interrupted recording or incomplete trial). The total number of such cases did not exceed one per test, and these exclusions did not affect group balance or statistical power.

## 5. Conclusions

The manuscript at hand summarizes the research on deciphering the properties of a virtually derived hit–SERAAK2. Primarily, in silico modeling confirmed that this ligand engages in interactions with conserved residues of 5-HT receptors (namely, 5-HT_1A_, 5-HT_2A_, 5-HT_7_); however, no meaningful interaction was detected for the dopamine D_2_ receptor.

ADMET studies revealed favorable characteristics, including the absence of CYP3A4 inhibition and moderate membrane permeability, although notable inhibition of CYP2D6 and concentration-dependent cytotoxicity deserve further optimization.

Behavioral studies in mice confirmed that SERAAK2 exerts anxiolytic and antidepressant effects at doses devoid of motor improvement while lacking cognitive effects in the passive avoidance task. Taken together, these findings highlight SERAAK2 as a promising serotonergic ligand with potential utility for the treatment of anxiety- and depression-related disorders.

While encouraging, these results also underscore the need for additional investigations. Future work should aim to refine the compound’s selectivity, clarify its precise receptor-mediated mechanisms of action, and evaluate long-term safety in advanced models.

Overall, by integrating in silico, biochemical, and behavioral approaches, this study provides a rational foundation for a continued development of SERAAK2 as a candidate CNS-active agent.

## Figures and Tables

**Table 1:** The inhibitory effect of the SERAAK2 compound on CYP3A4 activity. The results are expressed as percentage inhibition following incubation with a 10 µM concentration, averaged from two separate experiments (*n* = 3 per experiment).

Examined Compound	CYP3A4 Activity Inhibition, Evaluated at the Concentration of 10 µM [% of Inhibition ± SEM]
Ketoconazole	100 ± 1
SERAAK2	0 ± 2

**Table 2:** Inhibitory activity of the test compound against CYP2D6 at 10 µM.

Examined Compound	CYP2D6 Activity Inhibition, Evaluated in the Concentration of 10 µM [% of Inhibition ± SEM]
Quinidine	100 ± 1
SERAAK2	90 ± 1

**Table 3:** Viability reduction in HepG2 and SH-SY5Y cell lines following treatment with test compounds at 30 µM.

Examined Compound	Percentage Reduction in the Number of Viable HepG2 Cells [Mean ± SEM]	Percentage Reduction in the Number of Viable SH-SY5Y Cells [Mean ± SEM]
SERAAK2	104 ± 1	108 ± 1
DOX	100 ± 1	102 ± 1

**Table 4:** PAMPA permeability coefficients (P_e_).

Examined Compound	P_e_ ± SD [×10^−6^ cm/s]
Caffeine	12.4 ± 0.6
Sulpiride	0.011 ± 0.002
SERAAK2	1.10 ± 0.35

**Table 5:** Behavioral comparison of SERAAK1 and SERAAK2.

Test	SERAAK1	SERAAK2
Spontaneous locomotor activity	no change (30–60 mg/kg)	reduced activity at 15–60 mg/kg—effect likely due to CNS depression no effect at 7.5 mg/kg
Motor coordination (rotarod, chimney)	no impairment (30–60 mg/kg)—stable motor profile	impairment at 15–60 mg/kg no effect at 7.5 mg/kg—dose-dependent motor side effects
Elevated plus maze (EPM)	↑ open arm time and entries (30 mg/kg)—anxiolytic effect	↑ open arm time and entries (7.5 mg/kg)—anxiolytic effect
Forced swim test (FST)	↓ immobility (30 mg/kg)—antidepressant-like effect connected with serotonergic modulation	↓ immobility (7.5 mg/kg) antidepressant-like effect connected with serotonergic modulation
Passive avoidance (PA)	no data	no effect on memory consolidation
Amphetamine-induced hyperactivity	↓ hyperactivity (30 mg/kg)	no effect

## Data Availability

Data are available upon request.

## Supplementary Materials

The following supporting information can be downloaded at https://www.mdpi.com/article/10.3390/molecules30234633/s1, Figure S1. Molecular overlay of X-ray and docked poses in the redocking experiment.

## Abbreviations

The following abbreviations are used in this manuscript:

ADMET	Absorption, distribution, metabolism, excretion, and toxicity
EPM	Elevated plus maze test
FST	Forced swim test
GPCR	G protein-coupled receptor
MTS	3-(4,5-dimethylthiazol-2-yl)-5-(3-carboxymethoxyphenyl)-2-(4-sulfophenyl)-2H-Tetrazolium
PA	Passive avoidance test
PCA	Principal component analysis
PDB	Protein Data Bank
POPC	1-palmitoyl-2-oleoyl-sn-glycero-3-phosphocholine
RMSD	Root mean square deviation
RMSF	Root mean square fluctuation
SPC	Simple point charge
