# Supplementary material for: SERAAK2 as a Serotonin Receptor Ligand: Structural and Pharmacological In Vitro and In Vivo Evaluation
Source: Molecules. 2025 Dec 2;30(23):4633. doi: 10.3390/molecules30234633 (PMC12693327; doi:10.3390/molecules30234633)
Supplement: Supplementary file 1 [file molecules-30-04633-s001.zip › molecules-3954494-supplementary.pdf]

Supplementary Information

SERAAK2 as a Serotonin Receptor Ligand: Structural and Pharmacological In Vitro and In Vivo Evaluation

Agnieszka A. Kaczor [1,*], Agata Zięba [1], Tadeusz Karcz [2], Michał K. Jastrzębski [1], Katarzyna Szczepańska [3], Tuomo Laitinen [4], Marián Castro [5,6] and Ewa Kędzierska [7,*]

[1] Department of Synthesis and Chemical Technology of Pharmaceutical Substances with Computer Modeling Laboratory, Faculty of Pharmacy, Medical University of Lublin, 4A Chodźki St., PL-20093 Lublin, Poland; zieba.agata@gmail.com (A.Z.); michal.jastrz1998@gmail.com (M.K.J.)

[2] Department of Technology and Biotechnology of Drugs, Faculty of Pharmacy, Jagiellonian University Medical College, Medyczna 9, PL-30688 Cracow, Poland; t.karcz@uj.edu.pl

[3] Department of Medicinal Chemistry, Maj Institute of Pharmacology, Polish Academy of Sciences, 12 Smetna Str., 31-343 Krakow, Poland; k.szczep@if-pan.krakow.pl

[4] School of Pharmacy, University of Eastern Finland, Yliopistonranta 1, P.O. Box 1627, FI-70211 Kuopio, Finland; tuomo.laitinen@uef.fi

[5] Department of Pharmacology, Center for Research in Molecular Medicine and Chronic Diseases (CIMUS), Universidade de Santiago de Compostela, Avda de Barcelona, E-15782 Santiago de Compostela, Spain; marian.castro@usc.es

[6] Instituto de Investigación Sanitaria de Santiago de Compostela (IDIS), Travesía da Choupana s/n, E-15706 Santiago de Compostela, Spain

[7] Department of Pharmacology and Pharmacodynamics, Faculty of Pharmacy, Medical University of Lublin, 4A Chodźki St., PL-20093 Lublin, Poland

* Correspondence: agnieszka.kaczor@umlub.pl (A.A.K.); ewa.kedzierska@umlub.pl (E.K.)

Content

Figure S1. Molecular overlay of X-ray and docked poses in the redocking experiment.

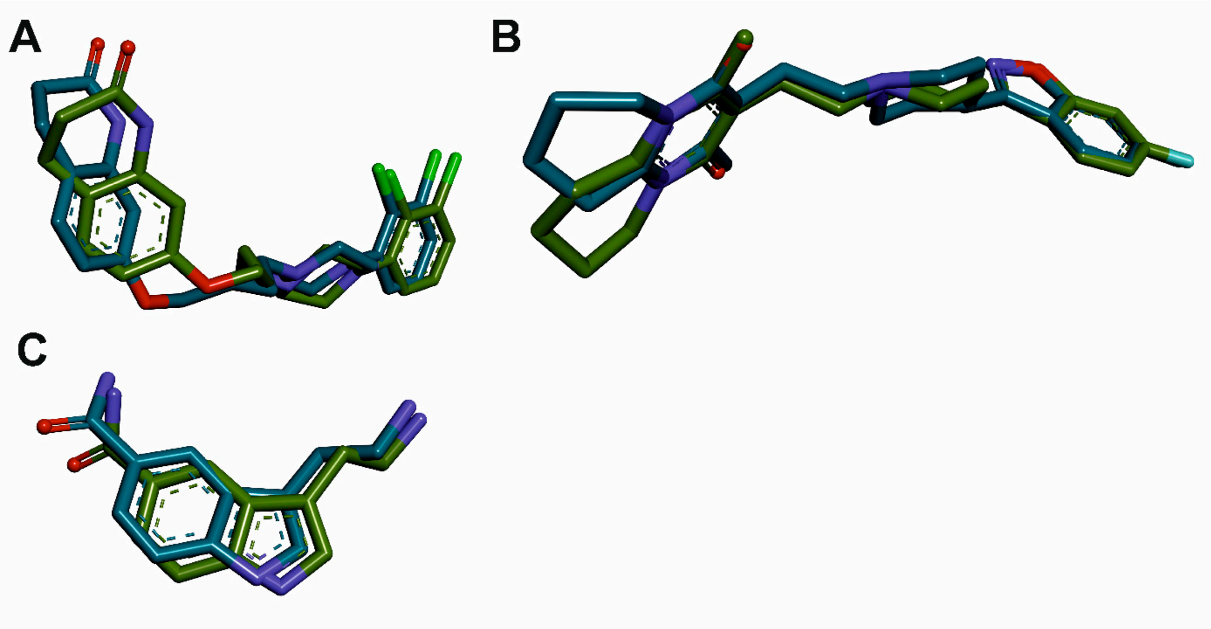

Figure S1. Molecular overlay of X-ray and docked poses in the redocking experiment for the 5-HT$_{1A}$ [PDB ID: 7E2Z] (A), 5-HT$_{2A}$ [PDB ID: 6A93] (B), and 5-HT$_{7}$ [PDB ID: 7XTC] (C) receptor ligands. X-ray molecules are shown with green carbon atoms and docked molecules with blue carbon atoms. Hydrogen atoms are omitted for clarity.
